# Centrosomes and cilia: always at the center of the action

**DOI:** 10.1038/s42003-020-01519-7

**Published:** 2020-12-14

**Authors:** Tiago J. Dantas

**Affiliations:** 1 i3S—Instituto de Investigação e Inovação em Saúde, Universidade do Porto, Porto, Portugal; 2 IBMC—Instituto de Biologia Molecular e Celular, Universidade do Porto, Porto, Portugal

## Abstract

Centrosomes are the main microtubule-organizing centers in animal cells, indispensable for cell division and the building of a wide range of cilia, which include sensory and motile cilia. We are now inviting submissions related to the fascinating field of centrosomes, cilia and all of the processes that they are involved in with the aim of highlighting this work in a Special Collection.

The term centrosome was first used by Theodor Boveri in the 19th century when referring to the *center body* inside the cell. Indeed, we now know that centrosomes have a central role in a vast range of cellular processes, which include mitosis, cytokinesis, ciliogenesis and cell migration. Most of these processes rely on the ability of centrosomes to function as the main microtubule-organizing centers (MTOCs)^1,2^.

“With your contribution, we believe that we can make this Collection a very useful resource to our community and deepen our understanding of the underlying causes of the disorders associated with centrosome and cilia dysfunction.”

Intense work over the last 30 years has helped unraveling the complexity of this organelle but many questions remain unanswered. At its core, each centrosome contains a pair of barrel-shaped, microtubule-based centrioles surrounded by a mesh of pericentriolar material (PCM) and centriolar satellites. It is the PCM that binds γ-tubulin ring complexes to nucleate and anchor the microtubule cytoskeleton. The interaction of centrosomes and their microtubule network with molecular motors allows the cell to strategically position them, for instance at the spindle poles during mitosis or in the leading process of a migrating neuronal precursor.

Each of the two centrioles at the center of the PCM is composed of nine microtubule triplets elegantly organized in nine-fold symmetry (Fig. 1a). The older, more mature centriole (generally referred to as “mother” centriole) has a central role in ciliogenesis as it serves as a nucleating basal body for all types of sensory and motile cilia. In fact, the presence of centrioles at the center of centrosomes is deeply interconnected with the ability of organisms to assemble cilia^2^ and species that do not make cilia have “centriole-less” centrosomes, such as yeast which have spindle pole bodies instead.

**Fig. 1:**
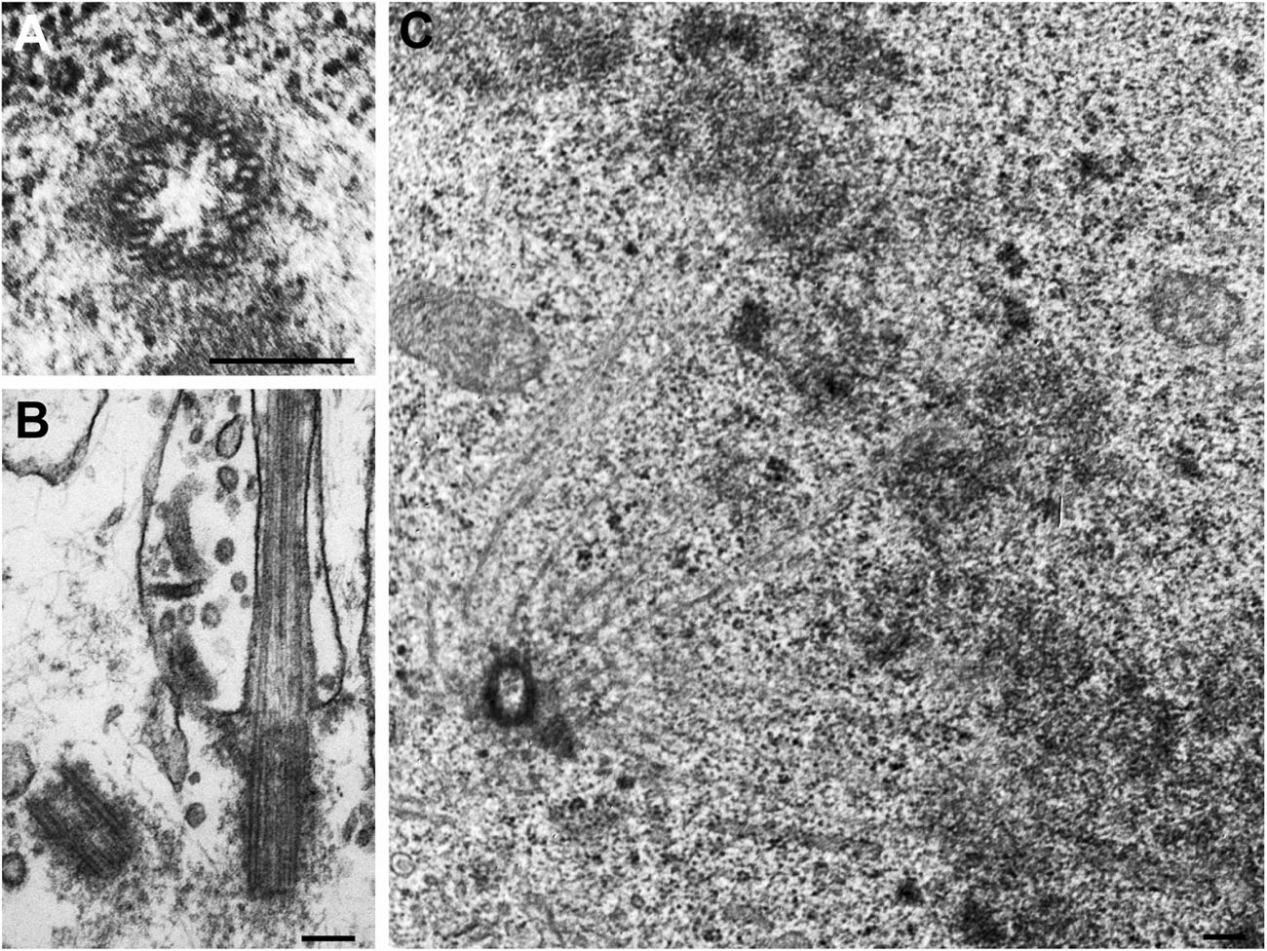
The ultrastructure of centrosomes and cilia. Transmission electron microscopy images of (**a**) the nine-fold symmetry of centriolar microtubule triplets in a human U2OS cell, (**b**) a primary cilium in a human RPE-1 cell and (**c**) a centrosome at one of the poles of a mitotic spindle in a chicken DT40 lymphocyte. Scale bars, 200 nm.

During the onset of ciliogenesis, the “mother” centriole uses specialized appendages at its distal end to attach to ciliary vesicles and to the plasma membrane. Microtubule doublets then extend from the centriole to form the ciliary axoneme (Fig. 1b), which functions as a highway for specialized molecular motors in a process known as intraflagellar transport (IFT). IFT is an important and very active area of research^1,3^ as it is essential for building cilia and for most ciliary functions. It is therefore not surprising that mutations in many genes encoding ciliary and IFT factors are associated with multi-system disorders commonly known as ciliopathies. The severe abnormalities associated with defects in cilia often include: neural tube and brain developmental defects, mental retardation, blindness, obesity, polydactyly and defects in the skeletal system, polycystic kidney and/or liver, infertility, *situs inversus* and airway infections^4^. These abnormalities in different organs reflect the wide range of cilia and their specialized functions in higher organisms^5,6^. However, more work is needed to further understand how mutations in genes encoding different subsets of centrosome/ciliary components lead to very different tissue abnormalities during development.

Interestingly, both cilia and centrosomes function as signaling hubs and are intrinsically associated with cell cycle progression, cell proliferation and differentiation^1,7^. In fact, defects in cilia disassembly and centrosome integrity result in a strong arrest at the G1 to S-phase transition^1^. On the other hand, the presence of supernumerary centrosomes is also associated with increased chromosome mis-segregation and cancer. Thus, the mechanisms controlling faithful centrosome duplication have been another topic of intensive research^1,2,8^. Now we understand that there are two main pathways/modes of producing centrioles/centrosomes: template-dependent centrosome duplication and de novo assembly. As implied by the name, the first pathway uses a preexisting centriole as a template to orthogonally assemble a nascent procentriole. This is a highly regulated pathway that ensures a tight control over centrosome number in proliferating cells. Conversely, the de novo pathway forms a high number of centrioles without the need of a template and is generally required for the assembly of high numbers of cilia in multiciliated cells, or in cells that completely lack centrioles.

Overall, despite recent insights into the sophisticated architecture of centrosomes and cilia, there is still much to learn about them. For instance, why does each centrosome have two centrioles rather than one, as these duplicate anyway once every cell cycle? Also, how do variations in the structure and composition of each type of cilia relate to their specialized functions in different cell types?

A parallel exciting area of research is the motor-dependent separation and alignment of centrosomes during the assembly of the bipolar spindle in mitosis^4^ (Fig. 1c). For example, spindle positioning is of particular importance for the proliferation of neural progenitors during brain development as the cell division axis is involved in fate determination. Clustering of centrosomes is also an important mechanism being studied as cancer cells with supernumerary centrosomes use it to form pseudo-bipolar spindles, reducing the rate of mitotic catastrophe induced by multipolarity. The active research of these topics is very promising for identifying and testing potential targets of novel chemotherapeutic drugs for cancer treatment.

## Call for papers on centrosomes and cilia for a Special Collection

The past years have yielded a significant improvement of our understanding of the key players and mechanisms regulating the biogenesis and functions of centrosomes and cilia. We at Communications Biology are very excited by the recent developments in the field, as illustrated by the papers we have already published on these topics^3,5–8^. To promote the efforts in advancing centrosome and cilia research, we decided to organize a Special Collection on these topics. We invite you to submit studies that dissect further the biogenesis and functions of centrosomes and cilia. We are also happy to receive studies focusing on all of the cellular and developmental processes that these remarkable organelles are involved in. We are confident that state-of-the-art imaging and biochemical techniques, will continue to unravel the interplay between the hundreds of components that integrate centrosomes and cilia-associated pathways. These approaches will also allow our community to better understand the surveillance mechanisms used by cells to monitor centrosome and cilia integrity/functions.

As an early career researcher at the Institute for Research and Innovation in Health (i3S) in Portugal, I am also aware of the challenges faced by my peers with regards to making our research visible to the larger community. As we develop this Centrosome and Cilia Collection at Communications Biology, we aim to be inclusive of exciting research from all career levels and hope to contribute to the exposure of young and rising investigators in the field. With your contribution, we believe that we can make this Collection a very useful resource to our community and deepen our understanding of the underlying causes of the disorders associated with centrosome and cilia dysfunction.
